# Primary anastomosis with diverting loop ileostomy vs. Hartmann’s procedure for acute diverticulitis: what happens after discharge? Results of a nationwide analysis

**DOI:** 10.1007/s00464-024-10752-8

**Published:** 2024-04-05

**Authors:** Arturo J. Rios Diaz, Lisa A. Bevilacqua, Theodore E. Habarth-Morales, Alicja Zalewski, David Metcalfe, Caitlyn Costanzo, Charles J. Yeo, Francesco Palazzo

**Affiliations:** 1 Department of Surgery, Thomas Jefferson University, Philadelphia, PA USA; 2 Sidney Kimmel Medical College, Thomas Jefferson University, Philadelphia, PA USA; 3 Oxford Trauma and Emergency Care, Nuffield Department of Orthopedics, Rheumatology and Musculoskeletal Sciences, University of Oxford, Oxford, UK

**Keywords:** Diverticulitis, Hartmann procedure, Diverting ileostomy, Hartmann reversal

## Abstract

**Background:**

Current guidelines recommend resection with primary anastomosis with diverting loop ileostomy over Hartmann’s procedure if deemed safe for acute diverticulitis. The primary objective of the current study was to compare the utilization of these strategies and describe nationwide ostomy closure patterns and readmission outcomes within 1 year of discharge.

**Methods:**

This was a retrospective, population-based, cohort study of United States Hospitals reporting to the Nationwide Readmissions Database from January 2011 to December 2019. There were 35,774 patients identified undergoing non-elective primary anastomosis with diverting loop ileostomy or Hartmann’s procedure for acute diverticulitis. Rates of ostomy closure, unplanned readmissions, and complications were compared. Cox proportional hazards and logistic regression models were used to control for patient and hospital-level confounders as well as severity of disease.

**Results:**

Of the 35,774 patients identified, 93.5% underwent Hartmann’s procedure. Half (47.2%) were aged 46–65 years, 50.8% female, 41.2% publicly insured, and 91.7% underwent open surgery. Primary anastomosis was associated with higher rates of 1-year ostomy closure (83.6% vs. 53.4%, *p* < 0.001) and shorter time-to-closure [median 72 days (Interquartile range 49–103) vs. 115 (86–160); *p* < 0.001]. Primary anastomosis was associated with increased unplanned readmissions [Hazard Ratio = 2.83 (95% Confidence Interval 2.83–3.37); *p* < 0.001], but fewer complications upon stoma closure [Odds Ratio 0.51 (95% 0.42–0.63); *p* < 0.001]. There were no differences in complications between primary anastomosis and Hartmann’s procedure during index admission [Odds Ratio = 1.13 (95% Confidence Interval 0.96–1.33); *p* = 0.137].

**Conclusion:**

Patients who undergo primary anastomosis for acute diverticulitis are more likely to undergo ostomy reversal and experience fewer postoperative complications upon stoma reversal. These data support the current national guidelines that recommend primary anastomosis in appropriate cases of acute diverticulitis requiring operative treatment.

**Graphical abstract:**

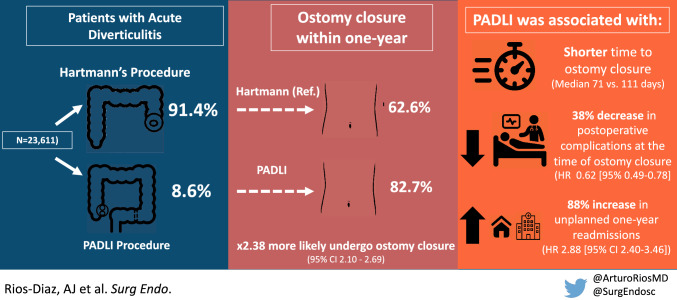

Colonic diverticular disease is highly prevalent in the American population; accounting for > 2.7 million outpatient visits and 200,000 inpatient admissions annually [1]. Over 50% of cases occur in individuals older than 60 years but the disorder increasingly affects younger people [1]. Around 25% of patients presenting in the emergent setting require surgical intervention [2], which has been historically a sigmoidectomy with end colostomy [Hartmann’s procedure (HP)]. However, subsequent stoma reversal can be challenging, resulting in a permanent colostomy in about 20–50% of these patients [3]. This has led to extensive debate about whether to pursue a partial colonic resection with primary colorectal anastomosis with diverting loop ileostomy (PADLI) in the emergent setting, which can be reversed more easily than an end colostomy.

There are concerns that primary anastomosis cannot be safely performed in a contaminated operative field, which is often a feature of perforated diverticulitis. However, many studies suggest that resection with primary anastomosis with or without a diverting stoma may be performed in clinically stable patients without significant comorbidities [4]. Even in patients with peritonitis or fecal contamination, PADLI can result in similar rates of morbidity and mortality compared to HP, but has been associated with shorter hospital stay and a higher rate of stoma reversal [5]. Others have suggested that PADLI confers a lower mortality rate [6, 7]. Nevertheless, national and international guidelines recommend performing HP in critically ill patients with multiple comorbidities [4, 8, 9].

The DIVERTI randomized controlled trial attempted to resolve controversies between PADLI and HP in terms of morbidity, mortality, and ostomy closure rates between these approaches for Hinchey Stage III–IV diverticulitis [2]. Although it showed no significant morbidity or mortality differences, the rate of stoma reversal was significantly higher after PADLI (96% vs 65%) by 18-months. Another study in the U.S. by Lee et al. using a large dataset reported similar overall morbidity between the two procedures but higher 30-day mortality associated with HP (7.6% vs 2.9%, *p* = 0.01) [7]. PADLI was not independently associated with any difference in the frequency of early postoperative complications or hospital length of stay (*p* = 0.05) [7]. However, this study cannot necessarily be generalized beyond centers participating in the National Surgical Quality Improvement Program (NSQIP) and outcomes beyond 30-days remain poorly understood.

The objective of the current study was to compare national ostomy closure and readmissions rates up to a year following urgent HP and PADLI for acute diverticulitis and the utilization of these surgical approaches at a national level. We hypothesized that the majority of patients undergo HP and that this procedure is associated with lower ostomy closure and higher readmissions than PADLI.

## Materials and methods

### Study design and data source

An observational cohort study was undertaken using the 2010–2019 Nationwide Readmission Database (NRD). The NRD is a publicly available de-identified Healthcare Cost and Utilization Project (HCUP) dataset comprised of data on all hospitalized patients in 27 contributing states. It contains over 100 clinical and nonclinical variables and can facilitate longitudinal follow-up of patients between hospitalizations within the same state. It can therefore track both planned and unplanned readmissions, even when they occur in different hospitals. Through sampling weights, the NRD can be used to generate national estimates [10]. The study design was deemed exempt from full review by the Thomas Jefferson University Institutional Review Board.

### Study population

The analysis included all adult patients (> 18 years) admitted between January 2010 and December 2019 with an *International Classification of Diseases, Ninth Revision, Clinical Modification* (ICD-9-CM) and *International Classification of Diseases, Tenth Revision, Clinical Modification* (ICD-10-CM) primary diagnosis code corresponding to diverticulitis of colon without mention of hemorrhage undergoing PADLI or HP. These procedures were defined using ICD-9-CM procedure codes and ICD-10-Procedural Coding Terminology (PCS) as described elsewhere (Appendix). [11] The exclusion criteria were trauma, malignancy, non-emergent/elective, inpatient mortality during index admission, ostomy closure during same admission, and transfers. In addition, patients with less than 6 months of follow-up data were excluded.

### Demographic, clinical, and hospital characteristics

Variables extracted included age, insurance, zip code, income quartile, same-state resident status, Elixhauser Comorbidity Index (ECI), NRD-provided comorbidities (chronic pulmonary disease, obesity, diabetes, hypertension, peripheral vascular disease, deficiency anemia, coagulopathy, weight loss), smoking, NRD-provided severity of illness, peritonitis, intraabdominal abscess, sepsis, laparoscopic approach, weekend admission, hospital colectomy volume quartile, hospital ownership, bed size of hospital (small, medium, and large, as defined by HCUP), teaching status, and hospital urban–rural designation.

### Outcomes

The primary endpoint was ostomy closure within 1 year of discharge as measure of “complete care” for acute diverticulitis that required colonic resection. Secondary endpoints included overall complications, non-surgery specific complications (cardiovascular, respiratory, genitourinary, thromboembolism), surgery-specific complications [gastrointestinal complications (gastric dilation, persistent postoperative vomiting, functional diarrhea, gastroenteritis and colitis, intussusception, ileus, volvulus, intestinal obstruction), delayed healing, surgical site infection, postoperative abscess, hematoma, seroma, disruption of wound, delayed healing, fistula, accidental puncture/injury, retained body, hemorrhage complicating procedure, blood transfusion, other postoperative complication (not classified elsewhere) and reoperation], non-routine discharge (discharge other than home), and prolonged length of stay (greater than > 75th percentile) during index admission and at the time of stoma closure. Other secondary endpoints included unplanned readmissions, overall readmissions (planned and unplanned), readmissions due to any complication, mortality upon readmission, incisional hernia (defined by diagnosis and or repair), and bowel obstruction after index procedure.

### Statistical analysis

Weighted proportions were used to report categorical variables and Pearson’s Chi-square tests for univariate comparisons. Hospital length of stay was right-skewed so reported as medians with interquartile range (IQR) and compared using linear combinations of parameters while taking into account the survey sampling weights. Kaplan–Meier plots were used to present ostomy closures at 12 months. Logistic regression and Cox proportional hazard models were fitted to adjust for confounders when comparing operative strategies. Potential confounders were identified a priori based on the literature [11–14], markers of severity of disease (peritonitis and NRD-provided severity of illness) and any variables associated with outcome (*p* < 0.2) in univariate analyses. The final covariates included in the regression models were: age, sex, insurance, income, weighted ECI, obesity, smoking status, severity of illness, peritonitis, laparoscopic approach, weekend admission, hospital volume, hospital teaching and rural status, and hospital bed size for all models. In addition, the ostomy closure and unplanned readmission models were fitted with non-routine discharge and inpatient complications at the time of index admissions as independent variables. The proportional hazards assumption was met for all Cox proportional hazards models. Factors associated with the primary outcome were also reported. In order to determine the impact of follow-up on the primary outcome, a sensitivity analysis was conducted comparing ostomy closure by group using a cohort of patients discharged in January to allow a uniform follow-up period (11–12 months). All models accounted for patient clustering within hospitals and obtained nationally weighted effects using the NRD-provided population design weights. A complete case approach was undertaken and records with missing data (2.9%) were excluded. The threshold for statistical significance was set a priori at two-tailed *p* < 0.05. All analyses were performed using StataMP v.17.0 (College Station, TX).

## Results

**Fig. 1:**
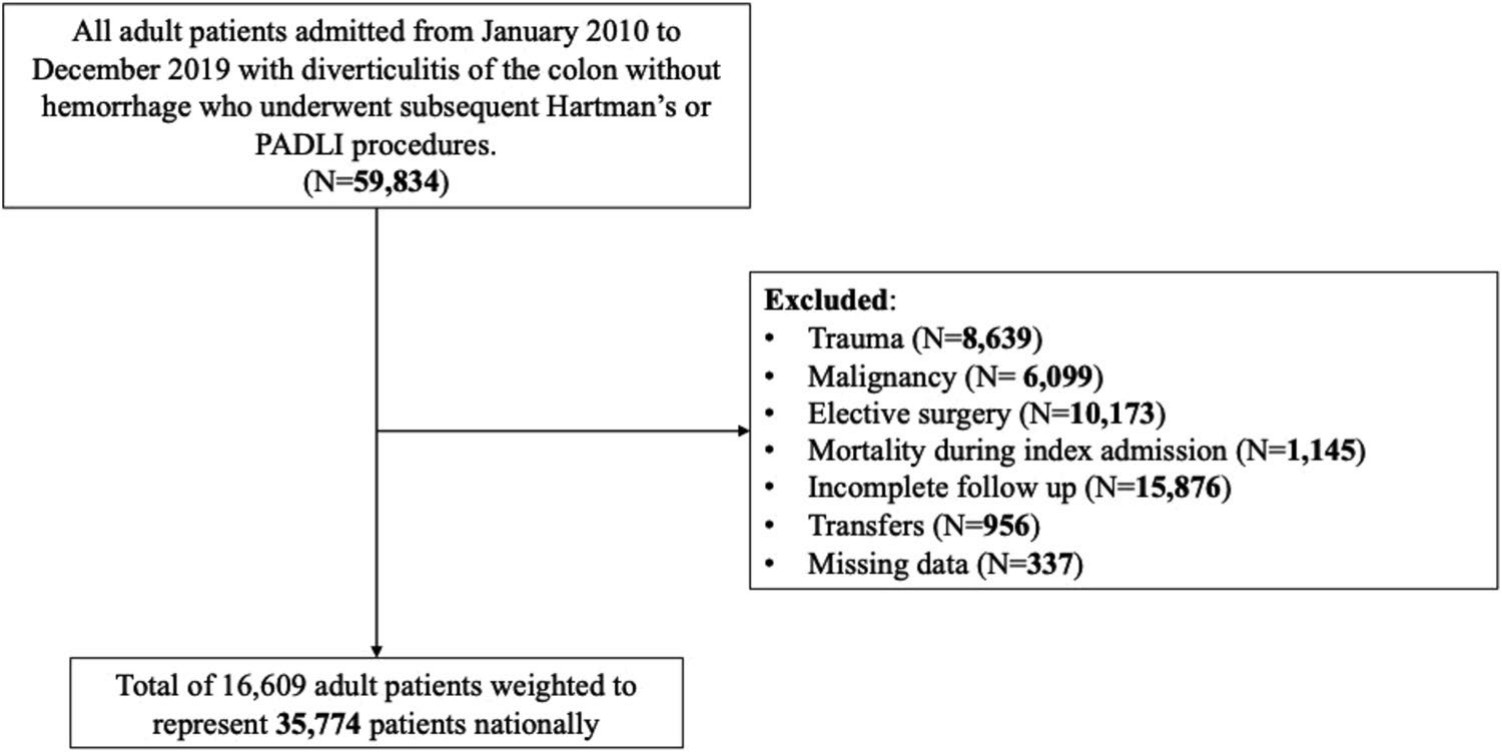
Flow diagram of inclusion and exclusion criteria

After inclusion and exclusion criteria were applied, there were 16,609 cases weighted to represent 35,774 nationally (Fig. 1). Most (93.5%) underwent Hartmann’s procedure. The majority were aged 61 years [median, IQR (51–71)], female (50.8%), publicly insured (Medicare 41.2%). The Hartmann group was more likely to be older and to have greater severity of illness (*p* < 0.001), peritonitis (23.9% vs. 16.1%; *p* < 0.001), and less likely to be admitted to a teaching (47.9% vs. 55.9%; *p* = 0.001) and large bed-size hospital (55.7% vs. 61.2%; *p* < 0.001) relative to the PADLI group (all *p* < 0.05; Table 1).

**Table 1:** Demographic, clinical mix, and hospital characteristics by management approach in a national sample of patients urgently admitted with acute diverticulitis undergoing PADLI or Hartmann’s procedure in the United States (weighted percentages)

	PADLI		Hartmann		Total		*p*
	(*N* = 2342)		(*N* = 33,433)		(*N* = 35,774)		
	*N*	%	*N*	%	*N*	%	
Age, year, median (IQR)	58.03	(51–70)	60.94	(50–69)	60.75	(51–71)	** < 0.001**
Female	1196	(51.1)	16,979	(50.8)	18,176	(50.8)	0.887
Insurance							**0.013**
Medicare	793	(33.9)	13,928	(41.7)	14,721	(41.2)	
Medicaid	199	(8.5)	2630	(7.9)	2829	(7.9)	
Private	1088	(46.5)	13,421	(40.1)	14,509	(40.6)	
Self-pay	144	(6.1)	2072	(6.2)	2216	(6.2)	
No charge/other	117	(5.0)	1382	(4.1)	1500	(4.2)	
Income quartile							0.407
0–25th percentile	513	(21.9)	8298	(24.8)	8811	(24.6)	
26th to 50th percentile	615	(26.3)	8792	(26.3)	9408	(26.3)	
51st to 75th percentile	625	(26.7)	8561	(25.6)	9186	(25.7)	
76th to 100th percentile	588	(25.1)	7782	(23.3)	8370	(23.4)	
Resident of same state	2241	(95.7)	31,831	(95.2)	34,072	(95.2)	0.646
Elixhauser comorbidity index score							**0.038**
0	399	(17.0)	4947	(14.8)	5346	(14.9)	
2-Jan	1068	(45.6)	14,306	(42.8)	15,374	(43.0)	
> 2	874	(37.3)	14,180	(42.4)	15,054	(42.1)	
Smoker	865	(36.9)	11,801	(35.3)	12,665	(35.4)	0.421
Obesity	425	(18.1)	5491	(16.4)	5916	(16.5)	0.271
Diabetes	224	(9.6)	3876	(11.6)	4100	(11.5)	0.108
Hypertension	1015	(43.4)	15,433	(46.2)	16,448	(46.0)	0.186
Peripheral vascular disorders	71	(3.0)	1381	(4.1)	1452	(4.1)	0.130
Chronic pulmonary disease	411	(17.6)	6279	(18.8)	6690	(18.7)	0.447
Deficiency anemia	28	(1.2)	624	(1.9)	651	(1.8)	0.179
Coagulopathy	59	(2.5)	906	(2.7)	964	(2.7)	0.750
Weight loss	384	(16.4)	3794	(11.3)	4178	(11.7)	** < 0.001**
Severity of illness (loss of function)							**0.068**
Minor	197	(8.4)	3364	(10.1)	3561	(10.0)	
Moderate	911	(38.9)	11,558	(34.6)	12,468	(34.9)	
Major	1016	(43.4)	14,539	(43.5)	15,555	(43.5)	
Extreme	218	(9.3)	3972	(11.9)	4191	(11.7)	
Peritonitis	377	(16.1)	7982	(23.9)	8359	(23.4)	** < 0.001**
Intraabdominal abscess	1228	(52.4)	12,587	(37.6)	13,815	(38.6)	** < 0.001**
Sepsis	13	(0.5)	999	(3.0)	1011	(2.8)	** < 0.001**
Laparoscopic approach	415	(17.7)	3073	(9.2)	3487	(9.7)	** < 0.001**
Hospital ownership							0.400
Government, nonfederal	238	(10.2)	3550	(10.6)	3789	(10.6)	
Private, not-profit	1793	(76.6)	24,867	(74.4)	26,661	(74.5)	
Private, invest-own	310	(13.2)	5015	(15.0)	5325	(14.9)	
Admission day is a weekend	525	(22.4)	8749	(26.2)	9274	(25.9)	**0.040**
Hospital colectomy volume quartile							**0.080**
1	346	(14.8)	6209	(18.6)	6555	(18.3)	
2	439	(18.8)	6447	(19.3)	6886	(19.3)	
3	369	(15.8)	5430	(16.3)	5799	(16.2)	
4	1187	(50.7)	15,317	(45.9)	16,504	(46.2)	
Teaching hospital	1308	(55.9)	16,038	(48.0)	17,346	(48.5)	**0.001**
Rural hospital	33	(1.4)	699	(2.1)	732	(2.0)	0.143
Hospital bed size							**0.017**
Small	379	(16.2)	5490	(16.4)	5869	(16.4)	
Medium	528	(22.5)	9320	(27.9)	9848	(27.5)	
Large	1435	(61.3)	18,623	(55.7)	20,058	(56.1)	

### Univariate analyses: outcomes during index admission and upon 1-year readmissions

The overall complication and non-surgery specific complication rates for the index admission were 49.3% and 23.5%, respectively, and did not differ between the groups (*p* > 0.005). Surgery-specific complications were higher in the PADLI group (41.2% vs. 37.1%, *p* = 0.041). Hartmann’s patients experienced more respiratory complications (8.3% vs. 5.9%, *p* = 0.036) but fewer accidental puncture/injuries (1.3 vs. 3.2%; *p* < 0.001) or surgical site infections (3.5% vs. 5.6%; *p* = 0.012; Table 2). Reoperations and non-routine discharge occurred in 1.7% and 71.8% of cases, respectively, but did not differ between the groups (*p* > 0.005). The overall length of stay was shorter for the Hartmann’s group [9 days (IQR 7–13)] compared to PADLI [10 days (IQR 7–15); *p* < 0.01], and the latter was more likely to experience prolonged length of stay (30% vs. 21.9%; *p* < 0.001).

**Table 2:** Index admission adverse outcomes in a national sample of patients urgently admitted with acute diverticulitis undergoing PADLI or Hartmann’s procedure in the United States (weighted percentages)

	PADLI		Hartmann		Total		*P*
	(*N* = 2342)		(*N* = 33,433)		(*N* = 35,774)		
	*N*	%	*N*	%	*N*	%	
Complication (any type)	1223	52.218	16,425	49.1274	17,647	49.3297	0.137
Non-surgical complication	535	22.8697	7888	23.5945	8424	23.5471	0.677
Cardiovascular complication	*	*	*	*	217	0.6073	**0.038**
Respiratory complication	138	5.8993	2761	8.2593	2899	8.1049	**0.036**
Renal complication	392	16.7275	5508	16.4759	5900	16.4923	0.868
Surgical complication (any)	964	41.1522	12,415	37.1335	13,378	37.3965	**0.041**
Surgical site infection	130	5.5614	1156	3.4567	1286	3.5945	**0.012**
Postoperative abscess	380	16.2415	4730	14.1482	5110	14.2852	0.152
Seroma	*	*	*	*	110	0.3084	0.678
Wound disruption	36	1.518	425	1.2722	461	1.2883	0.534
Fistula	*	*	*	*	12	0.0342	0.617
Accidental puncture	75	3.2036	437	1.308	512	1.4321	** < 0.001**
Retained foreign body	*	*	*	*	12	0.0344	0.338
Blood transfusion	251	10.7106	3101	9.2761	3352	9.37	0.251
Other postoperative complication, not otherwise classified	*	*	*	*	42	0.1168	0.715
Reoperation of abdomen^a^	46	1.9833	558	1.6682	604	1.6889	0.528
Non-routine discharge	1681	71.789	24,011	71.8171	25,692	71.8152	0.988
Prolonged length of stay (> 75th percentile)	700	29.8783	7306	21.8519	8005	22.3772	** < 0.001**

*Cells with counts equal or less than 10 were suppressed as per HCUP data user agreement

^a^Reoperation also included in the surgery-specific complication composite

**Fig. 2:**
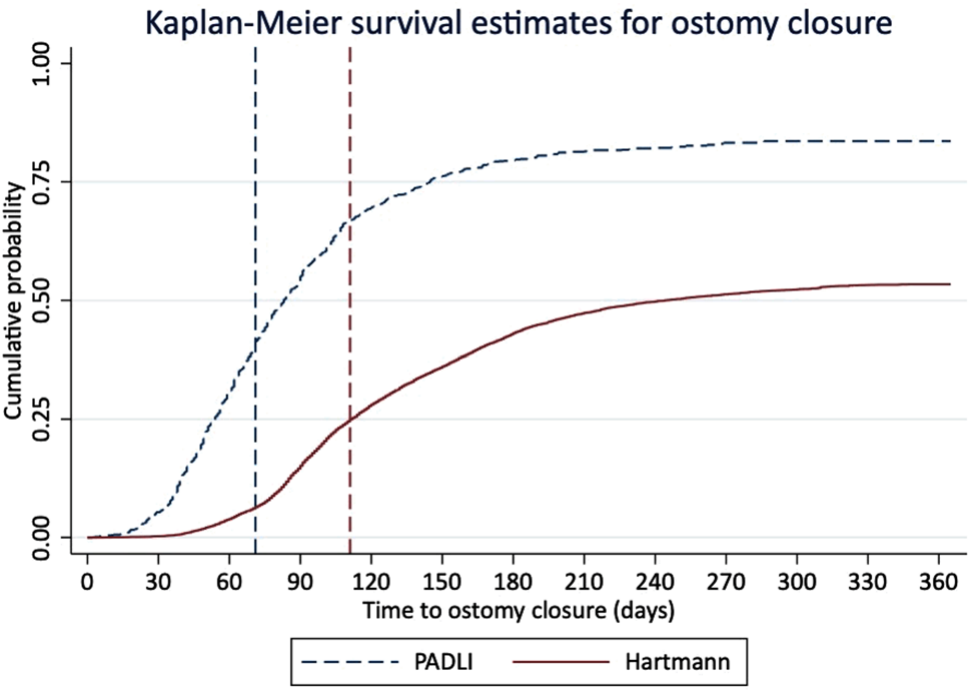
Reverse Kaplan–Meier curve showing the failure function curve for ostomy closure in a national sample of patients undergoing urgent PADLI vs. Hartmann’s for acute diverticulitis

The overall 1-year ostomy closure rate was 53.1%, which was significantly higher after PADLI (83.6% vs. 53.4%, *p* < 0.001; Fig. 2). The annualized number of patients who did not undergo stoma reversal in our cohort was 1901. These patients were more often older (64 vs. 58 years), publicly insured (59% vs 40%), in the bottom half of income quartiles (54.4 vs 47.2%) treated more often in out of state hospitals during their index procedure (93% vs 97%) in the bottom volume quartile centers (23.5% vs 14.7%), and had a greater comorbidity burden (ECI > 2: 50.6% vs 34.5%) compared to those who underwent closure within the study period (all *p* < 0.001). The median time from index hospital discharge to ostomy closure was significantly lower for PADLI patients [72 days (IQR 49–103) vs. 115 (86–160); *p* < 0.001]. In addition, overall (25.2% vs. 35.4%; *p* < 0.01) and surgery-specific (18.2% vs. 28%; *p* < 0.01) complications during ostomy closure were significantly lower after PADLI (data not shown in tables).

In terms of readmissions, patients who underwent PADLI had higher 1-year unplanned readmissions (29.8% vs. 20.0%; *p* < 0.001). Of these, 61.2% occurred within 30 days compared to 41.6% for HP (*p* < 0.001). Patients who underwent PADLI were also more likely to be readmitted due to a complication (22.8% vs. 16.8%; *p* < 0.001). Examination of selected conditions upon readmission revealed that bowel obstruction was more common in PADLI (16.7% vs. 13.1%, *p* = 0.012), incisional hernia more common after Hartman (11.8% vs. 5.5%, *p* < 0.001), and no difference in recurrent diverticulitis (2.6%, *p* = 0.804) (Table 3).

**Table 3:** Adverse outcomes upon 1-year readmissions in a national sample of patients urgently admitted with acute diverticulitis undergoing PADLI or Hartmann’s procedure in the United States (weighted percentages)

	PADLI		Hartmann		Total		*p*
	(*N* = 2342)		(*N* = 33,433)		(*N* = 35,774)		
	*N*	%	*N*	%	*N*	%	
Ostomy closure	1940	(82.9)	17,057	(51.0)	18,997	(53.1)	** < 0.001**
Readmission (any type)	2129	(90.9)	24,440	(73.1)	26,569	(74.3)	** < 0.001**
Unplanned readmission	698	(29.8)	6681	(20.0)	7380	(20.6)	** < 0.001**
Elective readmission	1431	(61.1)	17,735	(53.0)	19,166	(53.6)	** < 0.001**
All-cause mortality	*	*	*	*	219	(0.6)	0.443
Readmission due to any type of complication	533	(22.8)	5620	(16.8)	6153	(17.2)	** < 0.001**
Readmission due to recurrent diverticulitis	59	(2.5)	897	(2.7)	956	(2.7)	0.804
Reoperation	1969	(84.1)	15,610	(46.7)	17,579	(49.1)	** < 0.001**
Incisional hernia requiring repair	129	(5.5)	3943	(11.8)	4072	(11.4)	** < 0.001**
Bowel obstruction	391	(16.7)	4386	(13.1)	4777	(13.4)	**0.012**

*Cells with counts equal or less than 10 were suppressed as per HCUP data user agreement

### Multivariate analyses: ostomy closure, unplanned readmissions and complications

In multivariate analyses, PADLI was associated with increased ostomy closures [HR 2.46 (95% CI 2.19–2.75); *p* < 0.001] and with a 38% decrease in complications upon stoma closure [OR 0.51 (95% 0.42–0.63); *p* < 0.001]. PADLI was also associated with increased unplanned readmissions within 1 year [HR 2.83 (95% CI 2.38–3.37); *p* < 0.001]. There were no differences between surgical treatment management options in terms of complications during the index admission [PADLI HR 1.13 (95% CI 0.96–1.33); *p* = 0.137].

**Fig. 3:**
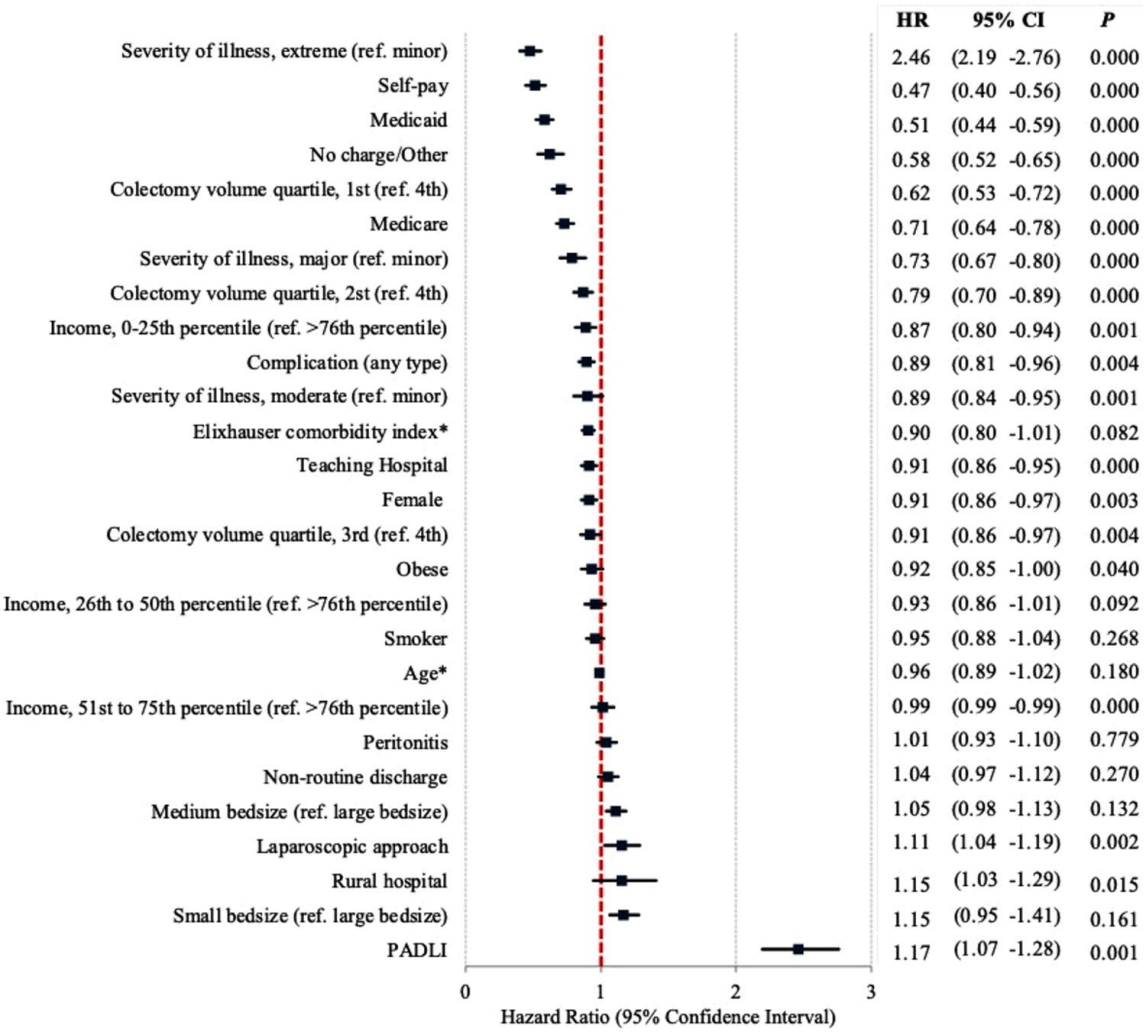
Forest plot depicting factors independently associated with ostomy closure within 1 year following urgent PADLI or Hartmann’s for acute diverticulitis

Within a Cox regression model, female patients, age, publicly insured and uninsured, those in the lowest income quartile, increased ECI, greater severity of illness and complications during the index admission, and those initially admitted to teaching and lower volume hospitals were less likely to undergo ostomy closure within 1 year. PADLI was the strongest predictor of ostomy closure at 1 year. Laparoscopic approach and admission to a small or medium-sized hospital were independently associated with increased ostomy closures within 1 year (Fig. 3).

## Discussion

This study used nationally representative longitudinal data to assess ostomy closure after urgent PADLI or Hartmann’s for patients admitted with acute diverticulitis. It found that PADLI patients had higher odds of ostomy closure within 1 year of discharge and fewer postoperative complications upon stoma closure. These findings are consistent with a number of small observational studies and the DIVERTI trial [2, 8, 15–18]. However, patients that underwent PADLI were more likely to experience 1-year unplanned readmissions. This study also highlights the underutilization of PADLI (< 9%) despite current Society of American Gastrointestinal and Endoscopic Surgeons (SAGES), American Society of Colon and Rectal Surgeons (ASCRS), and European Association of Endoscopic Surgery (EAES) guidelines preferring PADLI over HP procedure in appropriate cases. [8, 9]

The majority of patients in this study underwent ostomy closure within 1 year. The rate of ostomy reversal was higher for PADLI, which is in agreement with prior data [2, 19]. Although the absolute rate of reversal was much higher than previously reported using U.S. statewide data, the rate of 53.1% is congruent with rates reported in other studies [17, 19, 20]. Patients managed with PADLI were not only more likely to undergo ostomy closure but these procedures also occurred sooner than HP patients that underwent ostomy reversal. This may impact quality of life (QoL) and previous studies have reported that patients undergoing HP procedure have lower QoL than those undergoing PADLI [21]. The benefit on QoL may be related to higher and quicker reversal rates as the presence of a stoma was an independent predictor of worse QoL [21].

One disadvantage of PADLI in this study was the association with hospital readmissions. Unplanned readmissions were greater in the PADLI group, and the trend of high readmissions following ostomy creation has been described previously by other studies that examined readmissions following ileostomy [7, 22]. One of these studies did not find a significant difference between PADLI and Hartmann’s in terms of unplanned readmissions [7], however, the study was limited by a 30-day follow-up and only patients readmitted to the same hospital were captured. One explanation for this higher rate could be attributed to differences in operative approach. We found that there was a significant difference in laparoscopic approaches in the PADLI cohort. In a retrospective analysis of 603 PADLI procedures, laparoscopic approach was independently associated with 30-day readmissions, the majority of which were due to dehydration. Readmission is of importance not only from the policy and healthcare system perspectives, but it also can negatively impact QoL [23], limiting the perceived benefits of PADLI. Future trials should include a patient-centered outcome, such as QoL, and qualitative studies may help determine how patients prioritize higher likelihood of ostomy closure with the higher risk of having to be readmitted to hospital.

Post-operative complications were similar between the groups. These data suggest that PADLI and Hartmann’s procedure have similar safety profiles, both during the index admission and subsequently. This is consistent with prior studies that have assessed inpatient and 30-day postoperative outcomes [3, 5–7]. On the other hand, we found lower complications upon stoma reversal which is contrary to the DIVERTI trial [2] but in line with a prior study [19]. Reasons explaining this phenomenon and assessment of longitudinal outcomes following stoma closure warrant further research.

In terms of national practice patterns, over nine in every ten patients in this study were treated with Hartmann’s procedure. There are a number of possible explanations for this finding. Surgical dogma may impact decision making, despite recent studies showing that PADLI has higher rates of ostomy closure and a comparable safety profile [3, 5–7]. The Hartmann’s procedure had previously been the recommended procedure of choice for a long time and is more likely to be used by general surgeons relative to colorectal surgeons [24]. Furthermore, it provides shorter operative times by avoiding an anastomosis, and therefore may be the preferred option in critically ill and/or unstable patients in need of prompt source control as recommended by society guidelines [8, 9]. HP is also appropriate in circumstances when patients are at increased risk for anastomosis breakdown, such as those with immunocompromise [9]. Although our study did control for severity of illness as an important confounder, a more granular clinical assessment could not be performed due to the nature of the data used.

Another potential explanation for the practice pattern observed may be the late shift of guidelines toward PADLI. According to a prior systematic review of national and international society guidelines on the management of diverticulitis [25], only one society in The Netherlands recommended PADLI over Hartmann’s for hemodynamically stable patients by 2013. It was not until 2017 when a randomized control trial confirmed the safety of PADLI [2], which was followed by new recommendations published by SAGES and EAES in 2018 [8]. The findings of this study support these guidelines and provide more data to help guide surgical decision making.

### Limitations

The limitations of this study are largely related to its retrospective design using an administrative dataset. We relied on billing codes to characterize comorbidities and outcomes for which there is potential for selection and coding bias. In order to minimize misclassification bias, we used the NRD “chronic condition indicator” [26] to define new diagnoses as well as well as ICD-9-CM and ICD-10-CM codes related to complications of surgical and medical care. Another limitation is the inability to determine ostomy closure procedures that occurred beyond the calendar year. For this reason, we limited our cohort to those with at least a 6-month follow-up and conducted a sensitivity analysis only with patients with 11–12 months of follow-up and found the same trend favoring PADLI over Hartmann’s for ostomy closure. This dataset only captures hospitalizations that met inpatient criteria and therefore we were unable to account for ostomy closures that occurred in the ambulatory/outpatient setting (likely uncommon), as well as ED visits and observation stays for readmission analyses. However, given the high rates of ostomy closure and readmissions observed, it is likely most of them occurred in the inpatient setting. Lastly, we were unable to control for other socioeconomic determinants that may drive outcomes and readmissions, such as race. Nevertheless, this is unlikely to have affected our results as a prior study showed no differences in surgical treatment received or mortality for acute diverticulitis using nationwide data [27].

## Conclusion

Most patients with acute diverticulitis warranting surgical intervention undergo HP relative to PADLI in the U.S. During the first year after surgery, patients who undergo PADLI for acute diverticulitis are more likely to undergo ostomy reversal. These patients experience fewer postoperative complications upon stoma reversal but have higher unplanned readmissions after the index surgery despite shorter times to stoma reversal. Surgeons should be reassured that analysis of national data U.S. support the choice of PADLI over HP in the appropriate clinical setting for patients requiring urgent operative treatment for acute diverticulitis.

## Appendix

Definition criteria as defined by International Classification of Diseases, Ninth Revision, Clinical Modification (ICD-9-CM) codes.

**Appendix:** Definition criteria

Definition	ICD-9-CM procedure codes	ICD-10-PCS procedure codes
Partial colectomy with primary anastomosis and diverting loop ileostomy (PADLI)	Colonic resection (46.83, 17.35, 17.36, 45.75, 45.76) AND Ileostomy (46.20, 46.21, 46.22, 46.23, 46.01) Excluding patients with colostomy (46.11, 46.11, 46.13)	Colonic resection (0DB.PXXX, 0DB.NXXX, 0DB.MXXX, 0DB.LXXX, 0DB.GXXX, 0DB.EXXX, 0DT.PXXX, 0DT.NXXX, 0DT.MXXX, 0DT.LXXX, 0DT.GXXX, 0DT.EXXX) WITHOUT Colostomy formation (0D1.H0Z4, 0D1.H4Z4, 0D1.H8Z4, 0D1.K0Z4, 0D1.K4Z4, 0D1.K8Z4, 0D1.L0Z4, 0D1.L4Z4, 0D1.L8Z4, 0D1.N0Z4, 0D1.N4Z4, 0D1.N8Z4, 0D1.M074, 0D1.M0J4, 0D1.M0K4, 0D1.M0Z4, 0D1.M3J4, 0D1.M474, 0D1.M4J4, 0D1.M4K4, 0D1.M4Z4, 0D1.M874, 0D1.M8J4, 0D1.M8K4, 0D1.M8Z4, 0D1.N074, 0D1.N0J4, 0D1.N0K4, 0D1.N0Z4, 0D1.N3J4, 0D1.N474, 0D1.N4J4, 0D1.N4K4, 0D1.N4Z4, 0D1.N874, 0D1.N8J4, 0D1.N8K4, 0D1.N8Z4, 0D1.L074, 0D1.L0J4, 0D1.L0K4, 0D1.L0Z4, 0D1.L3J4, 0D1.L474, 0D1.L4J4, 0D1.L4K4, 0D1.L4Z4, 0D1.L874, 0D1.L8J4, 0D1.L8K4, 0D1.L8Z4) AND Ileostomy formation (0D1.90Z4, 0D1.94Z4, 0D1.98Z4, 0D1.A0Z4, 0D1.A4Z4, 0D1.A8Z4, 0D1.B8Z4, 0D1.B8Z4, 0D1.B4Z4, 0D1.B0Z4)
Hartmann procedure (HP)	Anterior resection of the rectum and colostomy (48.62) OR Left-sided colon resection (17.35, 17.36, 45.75, 45.76), AND Colostomy formation (codes 46.1, 46.10, 46.11, and 46.13)	Colonic resection (0DB.PXXX, 0DB.NXXX, 0DB.MXXX, 0DB.LXXX, 0DB.GXXX, 0DB.EXXX, 0DT.PXXX, 0DT.NXXX, 0DT.MXXX, 0DT.LXXX, 0DT.GXXX, 0DT.EXXX) AND Colostomy formation (0D1.H0Z4, 0D1.H4Z4, 0D1.H8Z4, 0D1.K0Z4, 0D1.K4Z4, 0D1.K8Z4, 0D1.L0Z4, 0D1.L4Z4, 0D1.L8Z4, 0D1.N0Z4, 0D1.N4Z4, 0D1.N8Z4, 0D1.M074, 0D1.M0J4, 0D1.M0K4, 0D1.M0Z4, 0D1.M3J4, 0D1.M474, 0D1.M4J4, 0D1.M4K4, 0D1.M4Z4, 0D1.M874, 0D1.M8J4, 0D1.M8K4, 0D1.M8Z4, 0D1.N074, 0D1.N0J4, 0D1.N0K4, 0D1.N0Z4, 0D1.N3J4, 0D1.N474, 0D1.N4J4, 0D1.N4K4, 0D1.N4Z4, 0D1.N874, 0D1.N8J4, 0D1.N8K4, 0D1.N8Z4, 0D1.L074, 0D1.L0J4, 0D1.L0K4, 0D1.L0Z4, 0D1.L3J4, 0D1.L474, 0D1.L4J4, 0D1.L4K4, 0D1.L4Z4, 0D1.L874, 0D1.L8J4, 0D1.L8K4, 0D1.L8Z4)
Ostomy closure	46.50, 46.51, 46.52	0DQ.80ZZ, 0DQ.83ZZ, 0DQ.84ZZ, 0DQ.87ZZ, 0DQ.88ZZ, 0DQ.90ZZ, 0DQ.93ZZ, 0DQ.94ZZ, 0DQ.97ZZ, 0DQ.98ZZ, 0DQ.A0ZZ, 0DQ.A3ZZ, 0DQ.A4ZZ, 0DQ.A7ZZ, 0DQ.A8ZZ, 0DQ.B0ZZ, 0DQ.B3ZZ, 0DQ.B4ZZ, 0DQ.B7ZZ, 0DQ.B8ZZ, 0DQ.8XXX, 0DQ.9XXX, 0DQ.AXXX, 0DQ.BXXX, 0D1.8XXX, 0D1.9XXX, 0D1.AXXX, 0D1.BXXX, 0WU.F0JZ, 0WU.F07Z, 0WQ.F0ZZ, 0WQ.FXZ2, 0WQ.FXZZ
